# Retraction: *In silico* 3D structure analysis accelerates the solution of a real viral structure and antibodies docking mechanism

**DOI:** 10.3389/fmicb.2015.01138

**Published:** 2015-10-20

**Authors:** Frontiers in Microbiology Editorial Office

The authors and the journal wish to retract the 6 November 2012 article cited above.

Based on information discovered after publication and reported to the journal in November 2014, this article was found to contain sections that were taken verbatim from other sources without giving proper reference to the source and without identifying the citation as a word-by-word citation from that source. In addition, credit was not given to all authors of the research reported in the article and copyright permissions were not obtained for the use of Figure 1. The authors agree with the retraction of the article and apologize to the readers, reviewers and editors of Frontiers in Microbiology.

## Conflict of interest statement

The authors declare that the research was conducted in the absence of any commercial or financial relationships that could be construed as a potential conflict of interest.

